# Estimating the purebred-crossbred genetic correlation of body weight in broiler chickens with pedigree or genomic relationships

**DOI:** 10.1186/s12711-019-0447-9

**Published:** 2019-02-19

**Authors:** Pascal Duenk, Mario P. L. Calus, Yvonne C. J. Wientjes, Vivian P. Breen, John M. Henshall, Rachel Hawken, Piter Bijma

**Affiliations:** 1 Animal Breeding and Genomics, Wageningen University and Research, P.O. Box 338, 6700 AH Wageningen, The Netherlands; 2 Cobb-Vantress Inc., Siloam Springs, AR 72761-1030 USA

## Abstract

**Background:**

In pig and poultry breeding programs, the breeding goal is to improve crossbred (CB) performance, whereas selection in the purebred (PB) lines is often based on PB performance. Thus, response to selection may be suboptimal, because the genetic correlation between PB and CB performance ($r_{pc}$) is generally lower than 1. Accurate estimates of the $r_{pc}$ are needed, so that breeders can decide if they should collect data from CB animals. $r_{pc}$ can be estimated either from pedigree or genomic relationships, which may produce different results. With genomic relationships, the $r_{pc}$ estimate could be improved when relationships between purebred and crossbred animals are based only on the alleles that originate from the PB line of interest. This work presents the first comparison of estimated $r_{pc}$ and variance components of body weight in broilers, using pedigree-based or genotype-based models, where the breed-of-origin of alleles was either ignored or considered. We used genotypes and body weight measurements of PB and CB animals that have a common sire line.

**Results:**

Our results showed that the $r_{pc}$ estimates depended on the relationship matrix used. Estimates were 5 to 25% larger with genotype-based models than with pedigree-based models. Moreover, $r_{pc}$ estimates were similar (max. 7% difference) regardless of whether the model considered breed-of-origin of alleles or not. Standard errors of $r_{pc}$ estimates were smaller with genotype-based than with pedigree-based methods, and smaller with models that ignored breed-of-origin than with models that considered breed-of-origin.

**Conclusions:**

We conclude that genotype-based models can be useful for estimating $r_{pc}$, even when the PB and CB animals that have phenotypes are closely related. Considering breed-of-origin of alleles did not yield different estimates of $r_{pc}$, probably because the parental breeds of the CB animals were distantly related.

**Electronic supplementary material:**

The online version of this article (10.1186/s12711-019-0447-9) contains supplementary material, which is available to authorized users.

## Background

In pig and poultry breeding programs, the breeding goal is to improve crossbred (CB) performance, whereas selection in the purebred (PB) lines is often based on PB performance. Thus, response to selection in CB performance may be suboptimal, because the genetic correlation between PB and CB performance ($r_{pc}$) is generally lower than 1 [1–3]. An $r_{pc}$ lower than 1 can be caused by genotype-by-environment interactions [4, 5], by genotype-by-genotype interactions in combination with allele frequency differences between the two parental breeds [6], and by differences in trait definitions between PB and CB performance [7, 8]. With a low $r_{pc}$, the use of CB instead of PB data may improve response to selection for CB performance [4, 9–11]. Thus, accurate estimates of the $r_{pc}$ are needed, so that breeders can decide if they should collect data from CB animals.

$r_{pc}$ is the additive genetic correlation between breeding values for PB and CB performance, and is defined as:

1 $$r_{{pc}} = \frac{{\sigma _{{A_{{PB,~CB}} }} }}{{\sigma _{{A_{{PB}} }} \sigma _{{A_{{CB}} }} }},$$

where $\sigma_{{A_{PB, CB} }}$ is the additive genetic covariance between breeding values for PB and CB performance and $\sigma _{{A_{{PB}} }}$ ($\sigma _{{A_{{CB}} }}$) is the additive genetic standard deviation in purebreds (crossbreds) [6, 12]. To estimate $r_{pc}$, phenotypic data from both PB and CB animals are needed. When these data are available, $r_{pc}$ can be estimated with a pedigree-based animal or sire model [13]. Such models treat PB and CB performance as correlated traits and use a pedigree-based relationship matrix (${\mathbf{A}}$) to link PB and CB observations [1]. To estimate $r_{pc}$ with ${\mathbf{A}}$, pedigree data should be available for both PB and CB individuals, and provide a link between PB and CB individuals. When the CB individuals are paternal half-sibs of the PB individuals, the accuracy of $r_{pc}$ estimated with ${\mathbf{A}}$ depends on the number of common sires between the PB and CB animals, and the accuracy of the estimated breeding values of the sires [14]. However, in practice, pedigree information is often not recorded in CB populations and the number of sires that have both PB and CB offspring with phenotypes may be limited.

These requirements for estimating $r_{pc}$ with pedigree information can be alleviated by replacing ${\mathbf{A}}$ with a multi-breed genomic relationship matrix (${\mathbf{G}}$) [15, 16]. An advantage of this approach is that the $r_{pc}$ can then also be estimated when the PB and CB animals are more distantly related, or when pedigree information is not recorded. In addition, genomic relationships may be more accurate than pedigree relationships [17, 18], which results in a smaller standard error of the estimate of $r_{pc}$ [19, 20].

Usually, the $r_{pc}$ between the CB and one of the PB parental lines is estimated. As such, genomic relationships between PB and CB animals should ideally be based on alleles that originate from that PB parental line only. However, the ordinary ${\mathbf{G}}$ is based on both alleles of an individual, which in the case of CB individuals, also include those originating from the other PB line. For example, when $r_{pc}$ is estimated between CB and its PB sire line, the ordinary ${\mathbf{G}}$ matrix is also based on alleles that originated from the dam line. An alternative for ${\mathbf{G}}$ is a genomic partial relationship matrix (${\mathbf{G}}_{BOA}$) that is based on the breed-of-origin of the alleles in the CB animals [21, 22]. Recently, a method to determine the breed-of-origin of alleles (BOA) based on phased genotypes was developed, allowing ${\mathbf{G}}_{BOA}$ to be constructed [23]. In ${\mathbf{G}}_{BOA}$, relationships between PB and CB animals are expected to be more accurate than in ${\mathbf{G}}$, because relationships in ${\mathbf{G}}_{BOA}$ are based on marker alleles that originated from the same breed. This approach was successfully applied to estimate variance components from data of three-way crossbred pigs, where 93% of the alleles of the crossbreds could be assigned a breed-of-origin [24, 25]. However, empirical studies in other species are lacking and, to date, no studies have compared $r_{pc}$ estimates and their standard errors from pedigree-based models to those from genotype-based models. In addition, it is not yet clear how $r_{pc}$ estimates and their standard errors are affected by the model used. Thus, our objective was to compare estimates of $r_{pc}$ and variance components obtained from pedigree-based and genotype-based models. In addition, we compared models that either consider or ignore breed-of-origin of alleles. We analysed body weight in broilers, using genotypes and measurements of PB and CB animals that have a common sire line.

## Methods

### Data

**Fig. 1:**
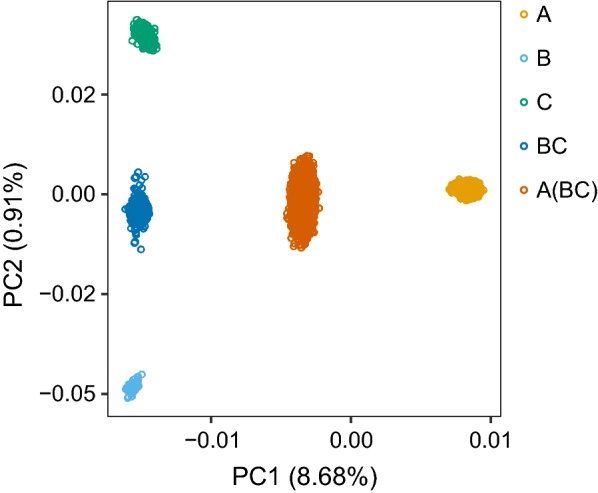
Principal component plot of the genotype data for the parental lines and the crossbreds. Values for principal component 1 (x-axis) are plotted against values for principal component 2 (y-axis). Between brackets is the variance explained by each principal component. Colours indicate genetic group

Data were collected on male and female broilers from a PB sire line (A) and on a three-way cross between this sire line and crossbred dams (BC), where lines B and C are dam lines. The dam lines were selected on egg production and the sire line on male fertility, along with standard traits, i.e., growth, yield, and feed efficiency. The three parental lines (A, B, and C) were genetically distant, as shown by the principal component analysis plot (Fig. 1). PB and CB animals were weighed between 6 and 8 days of age (BW7) and between 33 and 36 days of age (BW35). We chose these phenotypes because they are easy to measure proxies for growth, which is an important trait for breeding companies (Cobb; 2018 personal communication). Phenotype recording was done in five consecutive trials of similar size, which each included both PB and CB animals. All animals were housed in the same environment, in a barn located in Herveld, The Netherlands. The distribution of animals across trials and pens is in Table 1. Each pen had an approximately equal number of males and females. Offspring of a given sire were housed mostly in the same pen but each pen had offspring of multiple sires. Pens mostly had either PB or CB animals. An outlier analysis was done separately for PB and CB animals and separately for each day of measurement. Observations with standard deviations more than 3.5 away from the mean were considered as outliers and removed, which resulted in 4687 PB and 10,585 CB records on BW7 and 4471 PB and 10,272 CB records on BW35 (Table 2). The number of animals with observations ($N_{PB}$ for PB and $N_{CB}$ for CB animals) was smaller for BW35 than for BW7 because some animals did not survive until 35 days.

**Table 1:** Distribution of animals across trials and pens for body weight measured around 7 days (BW7)

Trial	Pen 1	Pen 2	Pen 3	Pen 4	NA^a^	Total
1	654	235	404	627	0	1920
2	821	0	829	860	0	2510
3	1281	1117	1122	1225	55	4800
4	1275	662	514	895	0	3346
5	1187	204	213	1092	0	2696
Total	5218	2218	3082	4699	55	15,272

The distribution of animals across trials and pens for body weight measured around 35 days (BW35) was very similar

^a^Number of animals with unknown pen

**Table 2:** Summary statistics for body weight measured around 7 (BW7) and 35 days (BW35)

	Number (N)	N sires	N dams	Mean	SD
BW7 (kg)					
Purebreds	4687	142	628	176	25
Crossbreds	10,585	156	1028	179	23
Total	15,272	161^a^	1656		
BW35 (kg)					
Purebreds	4471	140	623	2066	303
Crossbreds	10,272	156	1027	2090	302
Total	14,743	161^a^	1650		

Statistics are presented for PB and CB data, separately

*SD* standard deviations

^a^Total number of sires for all purebred and crossbred animals

All PB and CB animals with phenotypes were genotyped, as well as all their potential parents, and most of their potential grandparents. Markers with an unknown location, on sex chromosomes, on the mitochondrial genome, or with a call rate lower than 90% were removed. Marker positions were determined based on the *Gallus gallus* 4.0 (galGal4) reference assembly. All genotyped animals had a call rate of at least 90%. Genotypes were used for parentage assignment, such that pedigree information was available for all animals with phenotypes, up to the generation of their grandparents. The PB and CB animals had 161 unique PB sires, of which 135 sires had both PB and CB offspring with phenotypes, seven sires had only PB offspring with phenotypes, and 21 sires had only CB offspring with phenotypes (Table 2). The PB offspring had 628 unique dams, whereas the CB offspring had 1028 unique dams.

Markers with more than 1% inconsistent genotypes between derived parent–offspring pairs were removed and any remaining inconsistencies were set to missing. All missing genotypes of PB and CB animals were imputed simultaneously with FImpute [26]. Genotypes of the parents and grandparents were used to assign the breed-of-origin of alleles in the CB animals but were not included in the trait analyses. After assigning breed-of-origin, we removed markers if they had a minor allele frequency lower than 0.005 in either the genotype file or in the breed-of-origin file. These edits resulted in 50,960 markers that were used in the trait analyses.

### Assigning breed-of-origin of alleles

The breed-of-origin of alleles in the A(BC) crossbreds was derived with the BOA approach [23, 24]. In short, the BOA approach consists of (1) simultaneously phasing genotypes of PB and CB animals with AlphaPhase 1.1 by using pedigree information [27], (2) collecting a library of haplotypes for each line using phased haplotypes of the PB lines, and (3) assigning the breed-of-origin of alleles in the CB animals. With this approach, 49.5% of the alleles were assigned to sire line A, which is close to the expected 50%. The full procedure and results are described in Calus et al. [28].

### Statistical model

The BW7 and BW35 phenotypes were analysed separately with a bivariate model that treats PB and CB performance as separate but correlated traits. We compared four models that differed in the relationship matrix used. The general bivariate model can be written as [1, 29]:

2 $$\left[ {\begin{array}{*{20}c} {{\mathbf{y}}_{\text{PB}} } \\ {{\mathbf{y}}_{\text{CB}} } \\ \end{array} } \right] = \left[ {\begin{array}{*{20}c} {{\mathbf{X}}_{\text{PB}} } & 0 \\ 0 & {{\mathbf{X}}_{\text{CB}} } \\ \end{array} } \right]\left[ {\begin{array}{*{20}c} {{\mathbf{b}}_{\text{PB}} } \\ {{\mathbf{b}}_{\text{CB}} } \\ \end{array} } \right] + \left[ {\begin{array}{*{20}c} {{\mathbf{L}}_{{{\mathbf{PB}}}} } & 0 \\ 0 & {{\mathbf{L}}_{{{\mathbf{CB}}}} } \\ \end{array} } \right]\left[ {\begin{array}{*{20}c} {{\mathbf{m}}_{{{\mathbf{PB}}}} } \\ {{\mathbf{m}}_{{{\mathbf{CB}}}} } \\ \end{array} } \right] + \left[ {\begin{array}{*{20}c} {{\mathbf{Z}}_{\text{PB}} } & 0 \\ 0 & {{\mathbf{Z}}_{\text{CB}} } \\ \end{array} } \right]\left[ {\begin{array}{*{20}c} {{\mathbf{u}}_{\text{PB}} } \\ {{\mathbf{u}}_{\text{CB}} } \\ \end{array} } \right] + \left[ {\begin{array}{*{20}c} {{\mathbf{e}}_{\text{PB}} } \\ {{\mathbf{e}}_{\text{CB}} } \\ \end{array} } \right],$$

where ${\mathbf{y}}$ is a vector of phenotypes, ${\mathbf{b}}$ is a vector of fixed effects (breed × trial × pen × sex × age at measurement), with 85 (BW7) and 103 (BW35) levels, ${\mathbf{X}}$ is the design matrix of fixed effects, ${\mathbf{m}}$ is a vector of length equal to the total number of BC dams that contains (non-genetic) maternal effects with incidence matrix ${\mathbf{L}}$, ${\mathbf{u}}$ is a vector of length $\left( {N_{PB} + N_{CB} } \right)$ that contains additive genetic effects with incidence matrix ${\mathbf{Z}}$, and ${\mathbf{e}}$ is a vector of random residuals. Subscripts denote whether the terms relate to PB or CB performance. The distribution of maternal effects was $$\left[ {\begin{array}{*{20}c} {{\mathbf{m}}_{\text{PB}} } \\ {{\mathbf{m}}_{\text{CB}} } \\ \end{array} } \right]\sim\,N\left( {\left[ {\begin{array}{*{20}c} 0 \\ 0 \\ \end{array} } \right], \left[ {\begin{array}{*{20}c} {{\mathbf{I}}\sigma_{m,PB}^{2} } & 0 \\ 0 & {{\mathbf{I}}\sigma_{m,CB}^{2} } \\ \end{array} } \right]} \right),$$ where $\sigma_{m,PB}^{2}$ ($\sigma_{m,CB}^{2}$) is the maternal variance in the PB (CB) animals, and ${\mathbf{I}}$ is an identity matrix. Note that these maternal effects are not genetic effects, but permanent environmental effects. The distribution of additive genetic effects for PB (${\mathbf{u}}_{\text{PB}}$) and CB performance (${\mathbf{u}}_{\text{PB}}$) was:

3 $$\left[ {\begin{array}{*{20}c} {{\mathbf{u}}_{\text{PB}} } \\ {{\mathbf{u}}_{\text{CB}} } \\ \end{array} } \right]\sim\,N\left( {\left[ {\begin{array}{*{20}c} 0 \\ 0 \\ \end{array} } \right], \left[ {\begin{array}{*{20}c} {\sigma_{a, PB}^{2} } & {\sigma_{PB,CB} } \\ {\sigma_{PB,CB} } & {\sigma_{a, CB}^{2} } \\ \end{array} } \right] \otimes {\mathbf{K}}} \right),$$

where $\sigma_{a, PB}^{2}$ ($\sigma_{a, CB}^{2}$) is the additive genetic variance in the PB (CB) animals, $\sigma_{PB,CB}$ is the genetic covariance between PB and CB performance, and ${\mathbf{K}}$ is the relationship matrix between all animals, which differed between models. This parameterization yields additive genetic effects for both PB and CB performance of all animals. The distribution of residuals was $$\left[ {\begin{array}{*{20}c} {{\mathbf{e}}_{\text{PB}} } \\ {{\mathbf{e}}_{\text{CB}} } \\ \end{array} } \right]\sim\,N\left( {\left[ {\begin{array}{*{20}c} 0 \\ 0 \\ \end{array} } \right], \left[ {\begin{array}{*{20}c} {{\mathbf{I}}\sigma_{e,PB}^{2} } & 0 \\ 0 & {{\mathbf{I}}\sigma_{e,CB}^{2} } \\ \end{array} } \right]} \right)$$, where $\sigma_{e,PB}^{2}$ ($\sigma_{e,CB}^{2}$) is the residual variance in the PB (CB) animals. Concerning the fixed effects, we used the full interaction between effects (breed × trial × pen × sex × age at measurement), because males and females (in PB and CB animals) may have different growth rates (breed × sex × age at measurement), pens may have housed different groups of animals across trials (trial × pen), and the number of degrees of freedom (maximum 103) needed was acceptable for the size of this dataset.

Variance components were estimated by restricted maximum likelihood (REML) using the MTG2 software [30]. From the estimated variance components (indicated by ^), the estimate of $r_{pc}$ was computed as:

4 $$\hat{r}_{pc} = \frac{{\hat{\sigma }_{PB,CB} }}{{\hat{\sigma }_{a,PB} \hat{\sigma }_{a,CB } }}.$$

We compared estimates obtained from four models that use different relationship matrices, and we assessed model performance by comparing the standard errors and likelihoods of these models.

### Relationship matrices

We compared four models that use different relationship matrices (i.e., that replace ${\mathbf{K}}$ in Eq. (3): (1) based on pedigree (${\mathbf{A}}$; PED), (2) based on pedigree ignoring dams of CB animals (${\mathbf{A}}_{BOA}$; PED_BOA), (3) based on marker genotypes (${\mathbf{G}}$; GEN), and (4) based on marker alleles with sire origin (${\mathbf{G}}_{BOA}$; GEN_BOA). We included PED_BOA because it only fits the additive genetic effects for CB performance that are contributed by the sire line.

The ${\mathbf{A}}$ and ${\mathbf{A}}_{BOA}$ matrices were constructed from pedigree information, which was available for all animals with phenotypes, up to the generation of their grandparents. A single base population was assumed for all PB lines (i.e., no genetic groups were included). With ${\mathbf{A}}$, the full pedigree was used, whereas with ${\mathbf{A}}_{BOA}$, the dams of CB animals were set to missing. In addition, we set all the self-relationships of CB animals in ${\mathbf{A}}_{BOA}$ equal to 0.5 [31]. As such, PED_BOA is the pedigree equivalent of GEN_BOA. The ${\mathbf{G}}$ matrix was constructed following the multi-breed genomic relationship matrix of Wientjes et al. [16]:

5 $$\begin{aligned} {\mathbf{G}} & = \left[ {\begin{array}{cc} {{\mathbf{G}}_{\text{PB}} }  & {{\mathbf{G}}_{{{\text{PB}} - {\text{CB}}}} } \\ {{\mathbf{G}}_{{{\text{PB}} - {\text{CB}}}} }  & {{\mathbf{G}}_{\text{CB}} }  \\ \end{array} } \right] \\ & = \left[ {\begin{array}{cc} {\frac{{{\mathbf{M}}_{\text{PB}} {\mathbf{M}}_{\text{PB}}^{\prime } }}{{\sum 2p_{j}^{PB} \left( {1 - p_{j}^{PB} } \right)}}}  & {\frac{{{\mathbf{M}}_{\text{PB}} {\mathbf{M}}_{\text{CB}}^{\prime } }}{{\sqrt {\sum 2p_{j}^{PB} \left( {1 - p_{j}^{PB} } \right)} \sqrt {\sum 2p_{j}^{CB} \left( {1 - p_{j}^{CB} } \right)} }}} \\ {\frac{{{\mathbf{M}}_{\text{CB}} {\mathbf{M}}_{\text{PB}}^{\prime } }}{{\sqrt {\sum 2p_{j}^{PB} \left( {1 - p_{j}^{PB} } \right)} \sqrt {\sum 2p_{j}^{CB} \left( {1 - p_{j}^{CB} } \right)} }}}  & {\frac{{{\mathbf{M}}_{\text{CB}} {\mathbf{M}}_{\text{CB}}^{\prime } }}{{\sum 2p_{j}^{CB} \left( {1 - p_{j}^{CB} } \right)}}} \\ \end{array} } \right], \\ \end{aligned}$$

where ${\mathbf{M}}_{\text{PB}}$ (${\mathbf{M}}_{\text{CB}}$) is a centred marker genotype matrix of PB (CB) animals, and $p_{j}^{PB}$ ($p_{j}^{CB}$) is the allele frequency of marker $j$ in PB (CB) animals. We used the line-specific allele frequencies to separately centre the genotype matrices ${\mathbf{M}}_{\text{PB}}$ and ${\mathbf{M}}_{\text{CB}}$. The ${\mathbf{G}}_{BOA}$-matrix was constructed following Sevillano et al. [25] as:

6 $${\mathbf{G}}_{BOA} = \left[ {\begin{array}{*{20}c} {{\mathbf{G}}_{{BOA, {\text{PB}}}} } & {{\mathbf{G}}_{{BOA,{\text{PB}} - {\text{CB}}}} } \\ {{\mathbf{G}}_{{BOA, {\text{PB}} - {\text{CB}}}} } & {{\mathbf{G}}_{{BOA, {\text{CB}}}} } \\ \end{array} } \right] = \left[ {\begin{array}{*{20}c} {\frac{{{\mathbf{M}}_{\text{PB}} {\mathbf{M}}_{\text{PB}}^{\prime } }}{{\sum 2p_{j} \left( {1 - p_{j} } \right)}}} & {\frac{{{\mathbf{M}}_{\text{PB}} {\mathbf{T}}_{\text{CB}}^{\prime } }}{{\sum 2p_{j} \left( {1 - p_{j} } \right)}}} \\ {\frac{{{\mathbf{T}}_{\text{CB}} {\mathbf{M}}_{\text{PB}}^{\prime } }}{{\sum 2p_{j} \left( {1 - p_{j} } \right)}}} & {\frac{{{\mathbf{T}}_{\text{CB}} {\mathbf{T}}_{\text{CB}}^{\prime } }}{{\sum 2p_{j} \left( {1 - p_{j} } \right)}}} \\ \end{array} } \right],$$

where ${\mathbf{T}}_{\text{CB}}$ is a centred marker allele matrix of CB animals, with a value of $\left( {0 - p_{j} } \right)$ if the reference allele was inherited from the PB line, and a value of $\left( {1 - p_{j} } \right)$ if the alternative allele was inherited, where $p_{j}$ is the frequency of the alternative allele at marker $j$, which was calculated as the total number of alternative alleles in the PB and CB animals that were inherited from the PB line, divided by the total number of PB alleles in these animals. Note that the resulting ${\mathbf{G}}_{BOA}$ matrix is similar to the marker-based partial relationship matrix of Christensen et al. [22], with a scaling factor of $\sum 2p_{j} \left( {1 - p_{j} } \right)$.

The expected value of diagonal elements for CB animals in ${\mathbf{G}}_{BOA}$ and ${\mathbf{A}}_{BOA}$ is 0.5. The phenotypic variance of CB performance with PED_BOA and GEN_BOA was therefore computed as $0.5\sigma_{a,CB}^{2} + \sigma_{m,CB}^{2} + \sigma_{e,CB}^{2}$.

### Scaling of relationship matrices

With pedigree-based models, the population to which the variance components refer is the population of the founders of the pedigree. However, with genotype-based models, the reference population is, in most cases, the group of genotyped individuals, because ${\mathbf{G}}$ and ${\mathbf{G}}_{BOA}$ were constructed using the allele frequencies in the genotyped group. Thus, estimated variance components from pedigree- and genotype-based models are not directly comparable, because they refer to a different population [32]. To let the variance components from different models refer to the same (arbitrary) population, all relationship matrices were adjusted as:

$${\mathbf{K}}^{\prime } = \varvec{ }\left[ {\begin{array}{cc} {\frac{{{\mathbf{K}}_{11} }}{{D_{k1} }}} \hfill & {\frac{{{\mathbf{K}}_{12} }}{{\sqrt {D_{k1} } \sqrt {D_{k2} } }}} \hfill \\ {\frac{{{\mathbf{K}}_{21} }}{{\sqrt {D_{k1} } \sqrt {D_{k2} } }}} \hfill & {\frac{{{\mathbf{K}}_{22} }}{{D_{k2} }}} \hfill \\ \end{array} } \right],$$

where ${\mathbf{K}}_{11}$ denotes relationships among the PB animals, ${\mathbf{K}}_{22}$ denotes relationships among the CB animals, and ${\mathbf{K}}_{12}$ and ${\mathbf{K}}_{21}$ denote the relationships between PB and CB animals, as defined in Eqs. (5) and (6). Scalar $D_{k1}$ ($D_{k2}$) is the scaling factor of PB (CB) animals, which was defined as:

$$D_{{k_{x} }} = \overline{{Diag\left( {{\mathbf{K}}_{x} } \right)}} - {\bar{\mathbf{K}}}_{x} ,$$

where $\overline{{Diag\left( {{\mathbf{K}}_{x} } \right)}}$ is the mean of off-diagonals in ${\mathbf{K}}_{x}$ and ${\bar{\mathbf{K}}}_{x}$ is the mean of all elements in ${\mathbf{K}}_{x}$. This scaling procedure is equivalent to multiplying estimated variance components from models with unscaled relationship matrices by the appropriate scaling factors, as proposed by Legarra [32]. For models that considered the breed-of-origin of alleles, the expected value of $D_{{k_{2} }}$ was close to 0.5, so we used $2D_{{k_{2} }}$ instead of $D_{{k_{2} }}$ as a scaling factor in these models.

## Results

**Fig. 2:**
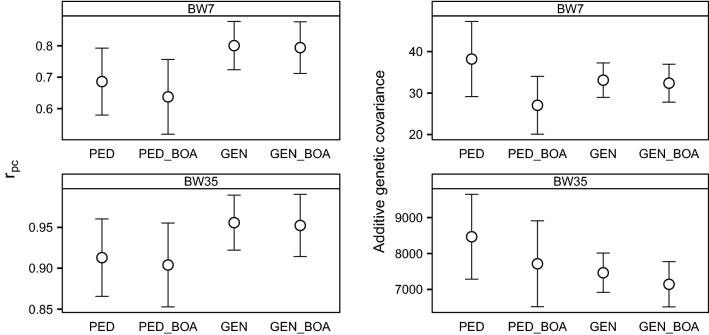
Estimates of $r_{pc}$ (left) and additive genetic covariance (right) from four models. Traits are body weight measured around 7 (BW7) and 35 days (BW35). Error bars represent standard errors reported by the MTG2 software

**Fig. 3:**
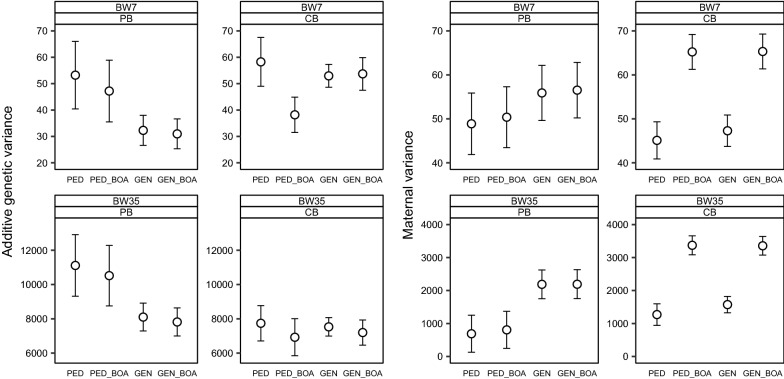
Estimates of additive genetic variance (left) and maternal variance (right) for purebred and crossbred body weight. Traits are body weight measured around 7 (BW7) and 35 days (BW35). Estimates are shown from four models. Error bars represent standard errors reported by the MTG2 software

Detailed information and estimates from all models are in Additional file 1. The phenotypic variance for BW7 was around 363 g^2^ for PB performance and 291 g^2^ for CB performance, whereas for BW35, it was around 37,048 g^2^ for PB performance and 33,455 g^2^ for CB performance. The estimated phenotypic variance was similar across models, thus we present variances instead of their ratio to phenotypic variance. Estimates of $r_{pc}$ and of the additive genetic covariance were larger for BW35 than for BW7 (Fig. 2). For BW7, the estimate of the additive genetic variance was smaller for PB performance than for CB performance, except with PED_BOA (Fig. 3). For BW35, estimates of the additive genetic variance and heritabilities were consistently larger for PB performance. Differences in estimates between models, were roughly similar for BW7 and BW35. For the sake of brevity, in the following, the description of results applies to both traits, unless stated otherwise. We will refer to PED and PED_BOA as pedigree-based models, and to GEN and GEN_BOA as genotype-based models. In addition, we will refer to PED and GEN as models that ignore breed-of-origin, and to PED_BOA and GEN_BOA as models that consider breed-of-origin.

### Pedigree versus genomic relationship information

Estimates of $r_{pc}$ were larger with genotype-based models than with pedigree-based models, particularly for BW7 (Fig. 2). However, estimates of the additive genetic covariance were smaller with genotype-based models than with pedigree-based models, except for BW7 and GEN_BOA versus PED_BOA. For PB performance, estimates of the additive genetic variance were smaller with genotype-based models than with pedigree-based models. For CB performance, estimates of the additive genetic variance were similar with genotype-based models and pedigree-based models, except for BW7 and GEN_BOA versus PED_BOA, for which the additive genetic variance was larger with GEN_BOA (Fig. 3). Because of these differences in estimates of additive genetic variance, the product of estimates of additive genetic standard deviations in the denominator of $r_{pc}$ was smaller with genotype-based models than with pedigree-based models. Estimates of the maternal variance of PB performance were larger with genotype-based models than with pedigree-based models, while for CB performance, estimates of maternal variance were similar for both types of models (Fig. 3).

### Ignoring versus considering breed-of-origin

Estimates of $r_{pc}$ from models that ignored or considered breed-of-origin were similar (Fig. 2). With pedigree-based models, estimates of the additive genetic covariance were smaller when breed-of-origin was considered, whereas with genotype-based models, estimates were similar. For PB performance, there were almost no differences in estimates of additive genetic variance and maternal variance (Fig. 3) between models that considered or ignored breed-of-origin. However, for CB performance, models that considered breed-of-origin had a larger estimate of maternal variance than models that did not. With pedigree-based models, the estimate of additive genetic variance of CB performance was smaller when breed-of-origin was considered than when it was not. However, with genotype-based models the estimate of additive genetic variance of CB performance was similar between models that considered or ignored breed-of-origin.

### Likelihoods and standard errors

For both traits, model GEN had the largest likelihood, followed by GEN_BOA, PED, and PED_BOA (Table 3). Likelihoods were larger for genotype-based methods than for pedigree-based methods, while considering breed-of-origin unexpectedly reduced likelihoods compared to ignoring breed-of-origin. In addition to the best fit, model GEN also gave the smallest standard error of estimates of $r_{pc}$, followed by GEN_BOA, PED, and PED_BOA (Fig. 2; Table 4). In general, the standard errors of estimates or $r_{pc}$ were smaller with genotype-based methods than with pedigree-based methods. The standard errors of estimates of variance components of CB performance were slightly larger with models that considered breed-of-origin compared to models that did not, while there were no differences in standard errors for estimates of variance components of PB performance (Fig. 3 and see Additional file 1).

**Table 3:** Likelihoods from four models for body weight measured around 7 (BW7) and 35 days (BW35)

Model	BW7		BW35	
	Likelihood	Relative to PED	Likelihood	relative to PED
PED	− 50,303.6064		− 83,374.8368	
PED_BOA	− 50,324.9275	− 21.321	− 83,397.1397	− 22.303
GEN	*− 50,143.4092*	160.197	*− 83,116.5065*	258.330
GEN_BOA	− 50,254.9253	48.681	− 83,275.2876	99.549

The likelihoods are those reported by MTG2. The largest likelihoods per trait are in italic. Models used a relationship matrix based on pedigree (PED), based on pedigree ignoring dams of CB animals (PED_BOA), based on marker genotypes (GEN), or based on marker alleles with sire origin (GEN_BOA)

**Table 4:** Estimates of the purebred-crossbred genetic correlation ($\varvec{r}_{{\varvec{pc}}}$) and their standard errors for body weight measured around 7 (BW7) and 35 days (BW35), from four models

Model	BW7		BW35	
	Estimate	Standard error	Estimate	Standard error
PED	0.69	0.11	0.91	0.05
PED_BOA	0.64	0.12	0.90	0.05
GEN	0.80	*0.08*	0.96	*0.03*
GEN_BOA	0.79	*0.08*	0.95	0.04

The standard errors are those reported by MTG2. The smallest standard errors per trait are in italic. Models used a relationship matrix based on pedigree (PED), based on pedigree ignoring dams of CB animals (PED_BOA), based on marker genotypes (GEN), or based on marker alleles with sire origin (GEN_BOA)

## Discussion

This study aimed at comparing models that estimate PB and CB genetic parameters of body weight in broiler chicken. We were particularly interested in the estimation of $r_{pc}$, because the value of $r_{pc}$ allows breeders to determine whether the use of CB information in the breeding program will increase genetic gain of CB performance, compared to a situation where only PB information is used. Our results showed that, for our population, $r_{pc}$ estimates were 5 to 25% larger with genotype-based models than with pedigree-based models. Moreover, $r_{pc}$ estimates were similar (max. 7% difference) with models that consider breed-of-origin and for models that ignore breed-of-origin. Genotype-based models had larger likelihoods and estimates with smaller standard errors than pedigree-based models, which was in line with expectations. This suggests that, although our results are not conclusive, $r_{pc}$ was underestimated with pedigree-based models in this study.

Estimates of $r_{pc}$ were between 0.64 and 0.80 for BW7 and between 0.90 and 0.96 for BW35. To our knowledge, this is the first time that $r_{pc}$ are estimated for body weight in broilers. It should be noted that, in this study, PB and CB animals were housed in the same environment. As such, our estimates provide an upper bound for values of $r_{pc}$ in situations where PB animals are housed in a breeding nucleus environment and CB animals in a commercial herd environment. Nevertheless, our estimates are similar to estimates from the literature on egg production traits in laying hens, for which estimates of $r_{pc}$ ranged from 0.62 to 0.83 [1]. In pigs, the average estimate of $r_{pc}$ for growth-related traits was lower (~ 0.6) [3].

With an $r_{pc}$ larger than ~ 0.7, the accuracy of predicting breeding values for CB performance is not expected to substantially improve when CB data instead of PB data is used [9]. An empirical study on pigs also showed that, with an $r_{pc}$ of about 0.90, replacing PB data with CB data did not improve prediction accuracy [33]. However, these results cannot be extrapolated directly to the current study, because differences in accuracy also depend on the number of phenotypic records available from the PB and CB populations and on the strength of relationships between the reference population and selection candidates [9, 19, 33]. In addition, information on PB performance may be more valuable than information on CB information, because the former may have been measured on the selection candidates themselves, whereas the latter can only be measured on relatives. Nevertheless, we expect that the use of CB instead of PB data will not substantially increase the accuracy of predicted breeding values for CB body weight in the current dataset, due to the high $r_{pc}$. A detailed investigation of the benefit of using CB instead of PB data for the accuracy of predicted breeding values will be investigated in a follow-up study.

Heritability estimates ranged from 0.09 to 0.20 for BW7 and from 0.21 to 0.30 for BW35. To our knowledge, heritability estimates for body weight at 7 days of age (BW7) have not been reported before. Our heritability estimates for BW35 were similar to those reported by Koerhuis and Thompson [34], Mulder et al. [35] and Maniatis et al. [36]. In contrast, our heritability estimates for BW35 were lower than those reported by Kapell et al. [37] and Rekaya et al. [38]. Estimates of the ratio of maternal to phenotypic variance ($m^{2}$) ranged from 0.13 to 0.22 for BW7 and from 0.02 to 0.10 for BW35. These results match with the general belief that maternal effects decrease with age. Estimates of $m^{2}$ for BW35 from the literature ranged from 0.02 to 0.05 [34, 36–38] and were somewhat smaller than our estimates, which may be due to the use of models that consider breed-of-origin in our study, where part of the genetic variance that is not captured moves to the non-genetic maternal variance.

### Pedigree versus genomic relationship information

Estimates of $r_{pc}$ were larger with genotype-based than with pedigree-based models, but the estimate of additive genetic covariance was often smaller with genotype-based models than with pedigree-based models, so the difference in $r_{pc}$ estimates was the result of differences in both additive genetic variances and covariance. The estimate of the additive genetic variance of PB performance was slightly larger with pedigree-based than with genotype-based models, while the estimate of the maternal variance of PB performance was smaller with pedigree-based models. First, the difference in variance estimates for PB performance may be due in part to bias in the genomic relationships that are estimated with markers [39]. To account for sources of bias when **G** is used, Goddard et al. [40] proposed to regress ${\mathbf{G}}$ towards ${\mathbf{A}}$. However, for our data, this procedure neither changed the relationships in ${\mathbf{G}}$ substantially, nor changed the estimates of variance components (results not shown). Furthermore, in contrast to PB performance, the additive genetic variance of CB performance was similar with pedigree-based and genotype-based models. Thus, we chose not to regress ${\mathbf{G}}$ towards ${\mathbf{A}}$. Second, the estimate of maternal variance may be more accurate with genotype-based than with pedigree-based models because genotype-based models may be more efficient at disentangling non-genetic maternal effects from the maternal component of an individual’s additive genetic effect [41, 42]. However, in contrast to PB performance, estimates of the additive genetic and maternal variances for CB performance were similar with pedigree-based and genotype-based models. Thus, it remains unclear why the differences in estimates of variances for PB performance between genotype-based and pedigree-based models were not observed for CB performance.

### The effect of considering breed-of-origin of alleles

For PB performance, estimates of variance components from models that ignored or considered breed-of-origin of alleles were similar, which is not surprising, because relationships between PB animals are the same regardless of whether breed-of-origin is considered or not. However, for CB performance, the estimate of the maternal variance was much larger with models that considered breed-of-origin. In these models, only alleles inherited from the sires were used to describe the variation in relationships between CB offspring. Thus, the genetic part of these models only captured the additive genetic variance of CB performance that is caused by the PB sire line. As a result, the non-genetic maternal effect absorbed most of the genetic variance caused by the BC dams. In contrast, with models that ignore breed-of-origin, alleles inherited from dams describe additional genetic covariation between CB offspring. Thus, the genetic components of these models also capture some of the additive genetic variance caused by the BC dams and, as a result, the variance explained by the maternal effect was smaller with models that ignored breed-of-origin than with models that considered breed-of-origin.

In spite of the differences in estimates of maternal variance between GEN and GEN_BOA, the estimate of additive genetic variance in CB performance ($\sigma_{a,CB}^{2}$) was similar between GEN and GEN_BOA. Thus, we hypothesized that either (1) the contribution of alleles that originated from the sire line to $\sigma_{a,CB}^{2}$ is equal to the contribution of alleles that originated from the BC dams, or (2) the relationships between the sires and BC dams contributed little to the estimate of $\sigma_{a,CB}^{2}$ (because the sires and BC dams were distantly related) and the paternal relationships dominated the estimate of $\sigma_{a,CB}^{2}$.

To test the first hypothesis, we analysed CB performance with a univariate model that fitted random sire and random dam effects separately, each with their own BOA matrix. This model yielded two estimates of $\sigma_{a,CB}^{2}$, one for the sire line and one for the BC dams, which showed that the contribution of the BC dams to the estimate of $\sigma_{a,CB}^{2}$ was larger than the contribution of the sire line (see Additional file 2). The first hypothesis was therefore rejected. To test the second hypothesis, we compared estimates of $\sigma_{a,CB}^{2}$ from the aforementioned univariate BOA approach with estimates from a univariate GEN approach using only CB performance (GEN_CB). With GEN_CB, the estimate of $\sigma_{a,CB}^{2}$ also depends on genetic covariances between sires and dams because GEN_CB merges alleles from both lines into a single ${\mathbf{G}}$ matrix. However, we observed that the average of the $\sigma_{a,CB}^{2}$ estimates of the sire line and BC dams from the BOA approach was close to the estimate of $\sigma_{a,CB}^{2}$ with GEN_CB (8389 vs 8410; see Additional file 2), which suggests that relationships between sires and dams contributed little to the likelihood or to the estimate of $\sigma_{a,CB}^{2}$ with GEN_CB. Indeed, there was almost no variance in genomic relationships between sires and dams and, as a result, relationships between sires and between dams dominated the estimate of $\sigma_{a,CB}^{2}$ with GEN_CB. Similar to $\sigma_{a,CB}^{2}$, the estimate of the additive genetic covariance between PB and CB performance was the same with GEN and GEN_BOA. Hence, the estimate of additive genetic covariance with GEN is probably dominated by variation in relationships between sires and between dams. Of these, we believe that paternal relationships dominated the estimate of $\sigma_{a,CB}^{2}$ because the model included a non-genetic maternal effect, which is strongly confounded with the maternal part of the genetic covariance between full sibs. Hence, covariances in the BC dams that are informative for $\sigma_{a,CB}^{2}$ originated mainly from more distant relationships, which have a smaller impact on the likelihood than, e.g., paternal half-sib relationships. In addition, the standard error of the estimate of $\sigma_{a,CB}^{2}$ was larger when using dam alleles than when using sire alleles (see Additional file 2), which suggests that paternal relationships dominated the estimate of $\sigma_{a,CB}^{2}$.

### Model usefulness

This study focused on the estimation of variance components and $r_{pc}$ using different models based on estimated standard errors and model fit. However, it should be noted that the model with the best fit does not necessarily yield the most accurate predicted breeding values [43], which shall be investigated in a follow-up study. Nevertheless, results showed that genotype-based models had a better model-fit and smaller estimated standard errors than pedigree-based models. Thus, genotype-based models may be preferred over pedigree-based models to estimate $r_{pc}$, even when the PB and CB animals are closely related. The benefit of genotype-based models may be slightly larger when the PB and CB animals are less related or when pedigree information is difficult to obtain. However, reported standard errors of estimates should be used with care. For example, the assumption in model GEN that all alleles in the CB animals originate from the same line is incorrect, which can lead to unreliable estimates of standard errors.

Models that consider breed-of-origin of alleles had smaller likelihoods than models that ignore it, which is somewhat unexpected for GEN versus GEN_BOA. With GEN, relationships between PB and CB animals are based on alleles from both the sires and dams and alleles of the dams in the PB and CB animals are assumed to have the same origin. Thus, the PB–CB relationships in ${\mathbf{G}}$ may be less accurate than the PB–CB relationships in ${\mathbf{G}}_{BOA}$, which may decrease the likelihood of GEN. Nevertheless, estimates of $r_{pc}$ with GEN and GEN_BOA were similar, which suggests that violation of model assumptions with GEN had only minor effects on the estimate of $r_{pc}$. In addition, GEN may have an advantage over GEN_BOA, because the assignment of the BOA is probably not without error, which may affect estimates of variance components.

The GEN_BOA model that we used in this study does not explicitly fit a genetic component for the maternal alleles in the CB animals and, hence, does not allow for a covariance of allele effects from the dams with those from the sire line. In addition, we were not able to estimate $r_{pc}$ between the A(BC) crossbreds and BC dams, because phenotypes of BC dams were not available. A more complete model would use phenotypes and phased genotypes from the A(BC) crossbreds and its three parental lines (A, B and C), model these phenotypes as four separate traits, and allow covariances between these traits [44]. Although such a model is more sophisticated and complete, we do not expect that it would result in different estimates of $r_{pc}$ between the CB and its sire line, because the parental lines were genetically distant.

In spite of differences in standard errors and likelihoods between models, we were not able to establish which estimates were closest to the true values (i.e., the genetic correlation at the causal loci) because this value is unknown. Gianola et al. [45] showed that estimates of genetic correlations using marker information may not necessarily reflect the true genetic correlation at causal loci because of imperfect linkage disequilibrium between markers and QTL. However, simulation studies have suggested that genotype-based models result in unbiased estimates of genetic correlations when relationships at causal loci are accurately predicted by the markers [46]. Further research is needed to establish whether these results also apply to estimation of $r_{pc}$ and which of the models presented in this study yields the most accurate estimate of $r_{pc}$.

## Conclusions

This work presents the first comparison of estimated $r_{pc}$ and variance components of body weight in broilers, using pedigree-based and genotype-based models, where the breed-of-origin of alleles was either ignored or considered. Estimates of $r_{pc}$ ranged from 0.64 to 0.80 for BW7 and from 0.90 to 0.96 for BW35. Genotype-based models resulted in larger estimates of $r_{pc}$ than pedigree-based models and are preferred for estimating $r_{pc}$ because they resulted in smaller standard errors of estimates and had better model fit than pedigree-based models. Considering breed-of-origin of alleles did not affect estimates of $r_{pc}$, probably because the parental breeds of the CB animals were distantly related but could result in different estimates of $r_{pc}$ when the parental breeds are more closely related, or when the amount of data is limited.

## Additional files

**Additional file 1.** Estimates of variance components and purebred-crossbred genetic correlations of body weight around 7 (BW7) and 35 days (BW35) for four models. Description: Estimates of variance components are presented for each purebred and crossbred trait. Column A indicates the parameter, and column B indicates the model used.

**Additional file 2.** Estimates of variance components for CB performance of BW35 from models that either fit a single G matrix (GEN_CB), or that separately fit a genetic sire component and a genetic dam component with two BOA matrices.
